# Supplementary material for: ETNK1 mutations induce a mutator phenotype that can be reverted with phosphoethanolamine
Source: Nat Commun. 2020 Nov 23;11:5938. doi: 10.1038/s41467-020-19721-w (PMC7684297; doi:10.1038/s41467-020-19721-w)
Supplement: Supplementary file 1 — Supplementary information [file 41467_2020_19721_MOESM1_ESM.pdf]

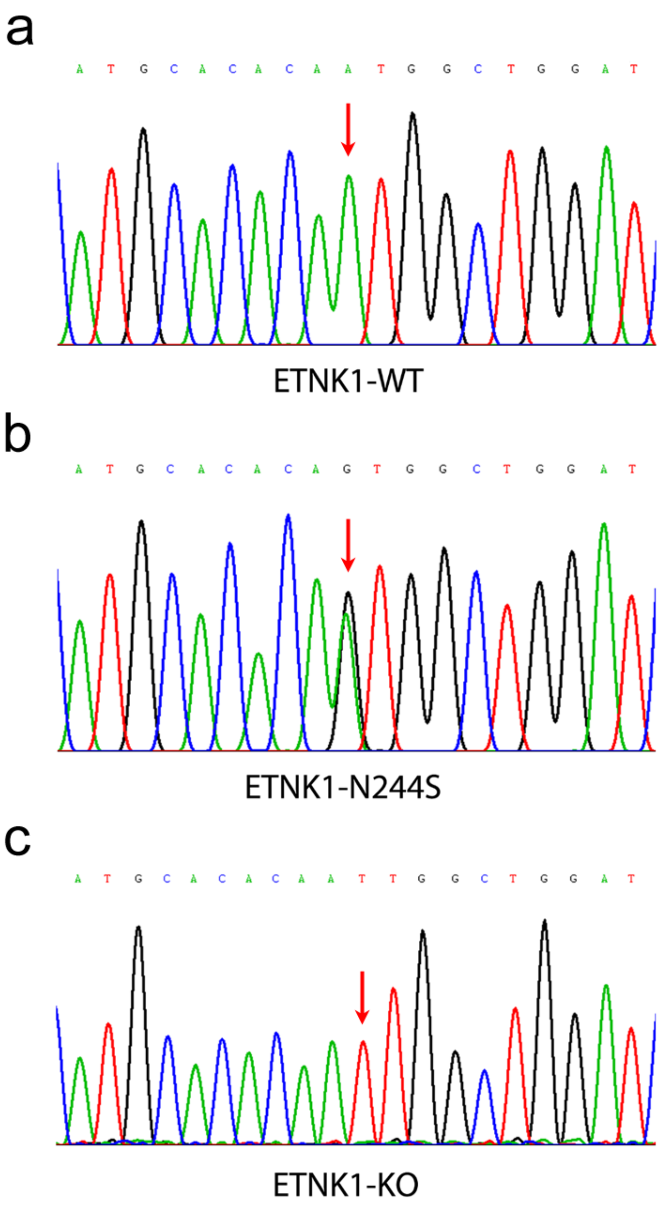

Supplementary Figure 1: Sanger sequencing. Detail of chromatograms regarding ETNK1-WT (A), ETNK1-N244S (B), and ETNK1-KO (C) loci. Wild-type A (upper panel), A to G substitution (middle panel) and Tins (bottom panel) are highlighted by a red arrow.

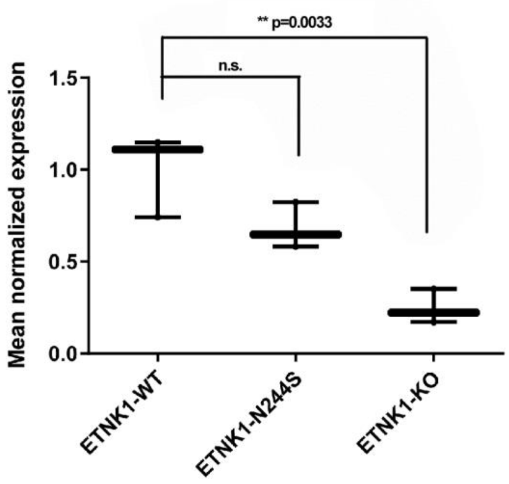

Supplementary Figure 2: ETNK1 mRNA expression. RT-qPCR analysis of total RNA extracted from ETNK1-WT, N244S, and KO cell lines, normalized on ETNK1-WT. The boxplots delimit the interquartile range; the central bar represents the median; the whiskers extend from minimum to maximum (n=3 independent samples). Statistical analyses were performed using one-way ANOVA with Tukey's post-hoc test. Source data are provided as a Source Data file.

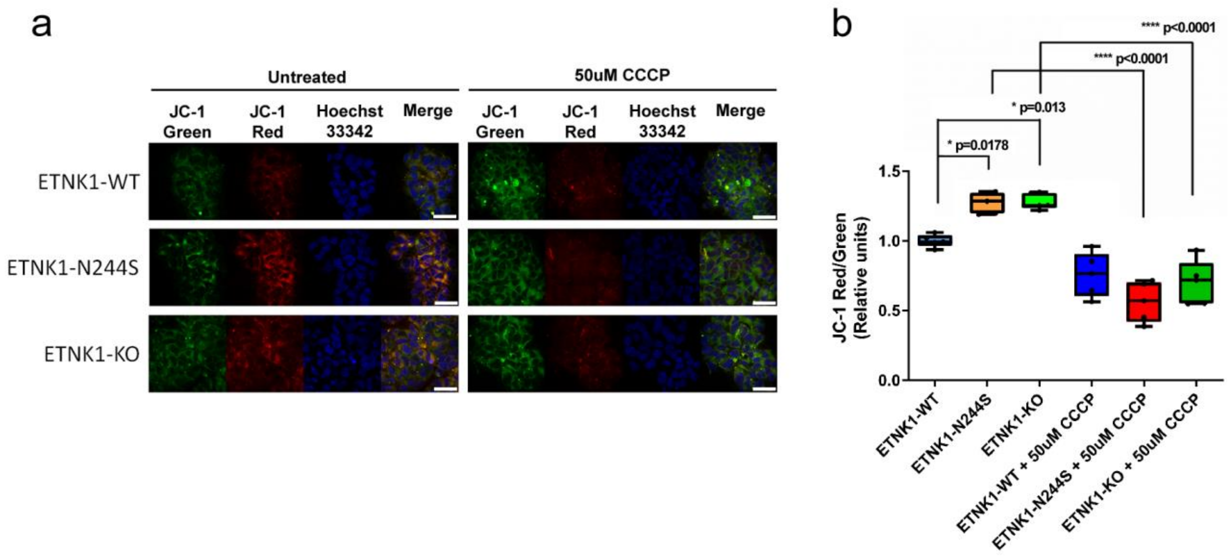

Supplementary Figure 3: Mitochondrial activity. A) Confocal microscopy of JC-1 red/green signal in ETNK1-WT, N244S, and KO cell lines in the absence (left) and presence (right) of 50µM CCCP. At least 200 cells were analyzed. The scale bar corresponds to 40µm. B) Boxplots showing the JC-1 red-to-green signal ratio of ETNK1-WT, N244S, and KO cell lines normalized on ETNK1-WT in the absence and presence of 50µM CCCP. The boxplots delimit the interquartile range; the central bar represents the median; the whiskers extend from minimum to maximum (n=5 representative fields). Statistical analyses were performed using one-way ANOVA with Tukey's post-hoc test. Source data are provided as a Source Data file.

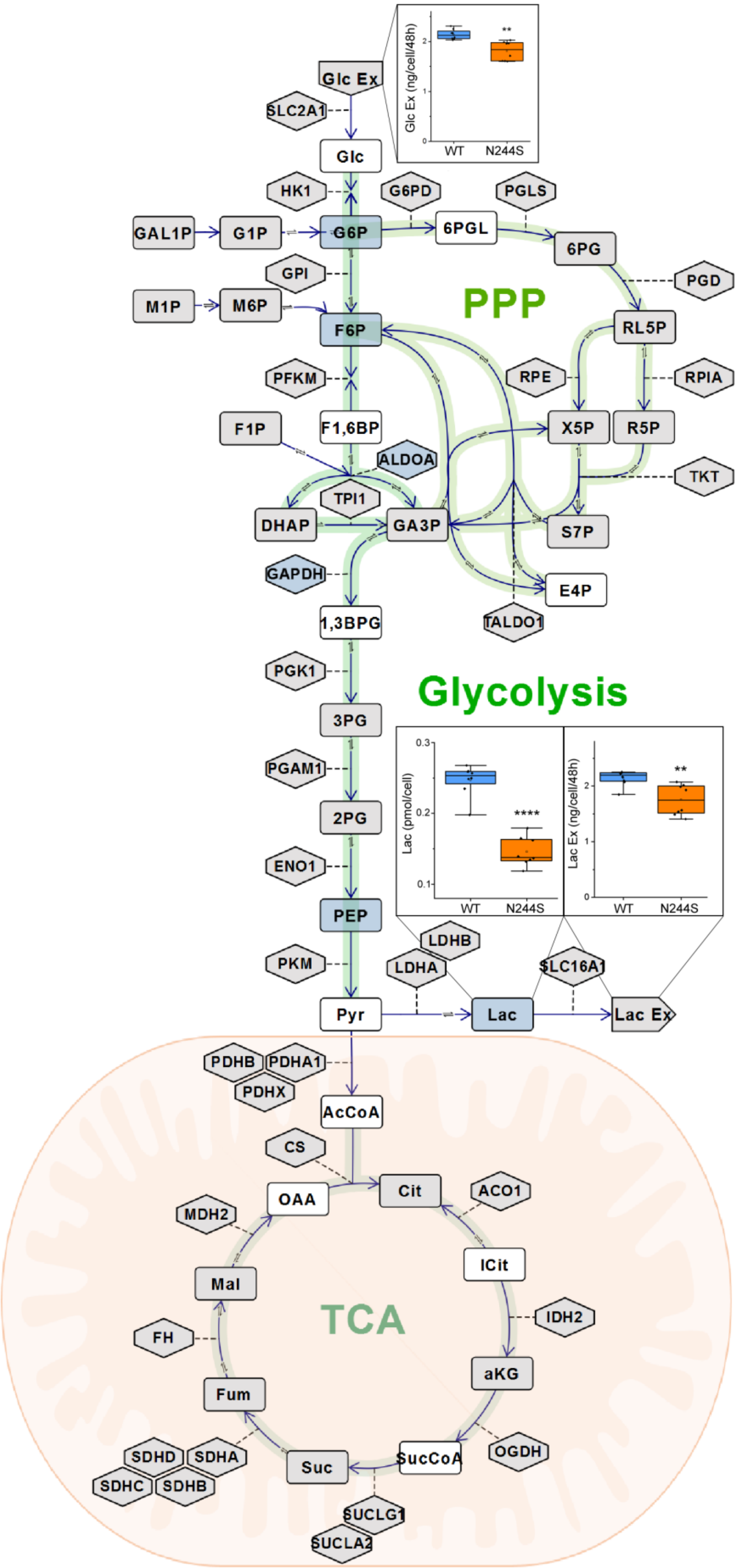

Log2 fold change colour scale:

x ≤ -1	-1 < x ≤ -0.5	-0.5 < x < -0.5	0.5 ≤ x < 1	x ≥ 1

Supplementary Figure 4: Central carbon metabolism. Simplified schematic overview of glycolysis, the pentose phosphate pathway (PPP), and the tricarboxylic acid (TCA) cycle showing metabolite levels and enzyme expression levels in ETNK1-N244S cell lines relative to ETNK1-WT cell lines. Data is in Log_2-space. Rectangular boxes represent metabolite levels as measured by capIC-MS/MS and LC-MS/MS, arrow-shaped boxes represent consumption or excretion/cell/48h as measured by NMR, and diamond shaped boxes represent enzyme expression levels as measured by RNA-Seq analysis. RNA/metabolite levels differing significantly between genotypes (unpaired, two-sided t-test) are shown as boxplots, with boxes representing the interquartile range, the central bar representing the median and the whiskers extending from minimum to maximum (n=8 biologically independent samples; p=0.0013 for glucose excretion; p<0.0001 for lactate concentration; p=0.0046 for lactate excretion).
Metabolites in white colour were not quantified. G1-/M1P, GA3P/DHAP, 2-/3PG, and R-/RL-/X5P could not be chromatographically separated, and are presented together. For abbreviations, see Supplementary Data 2. Source data are provided as a Source Data file.

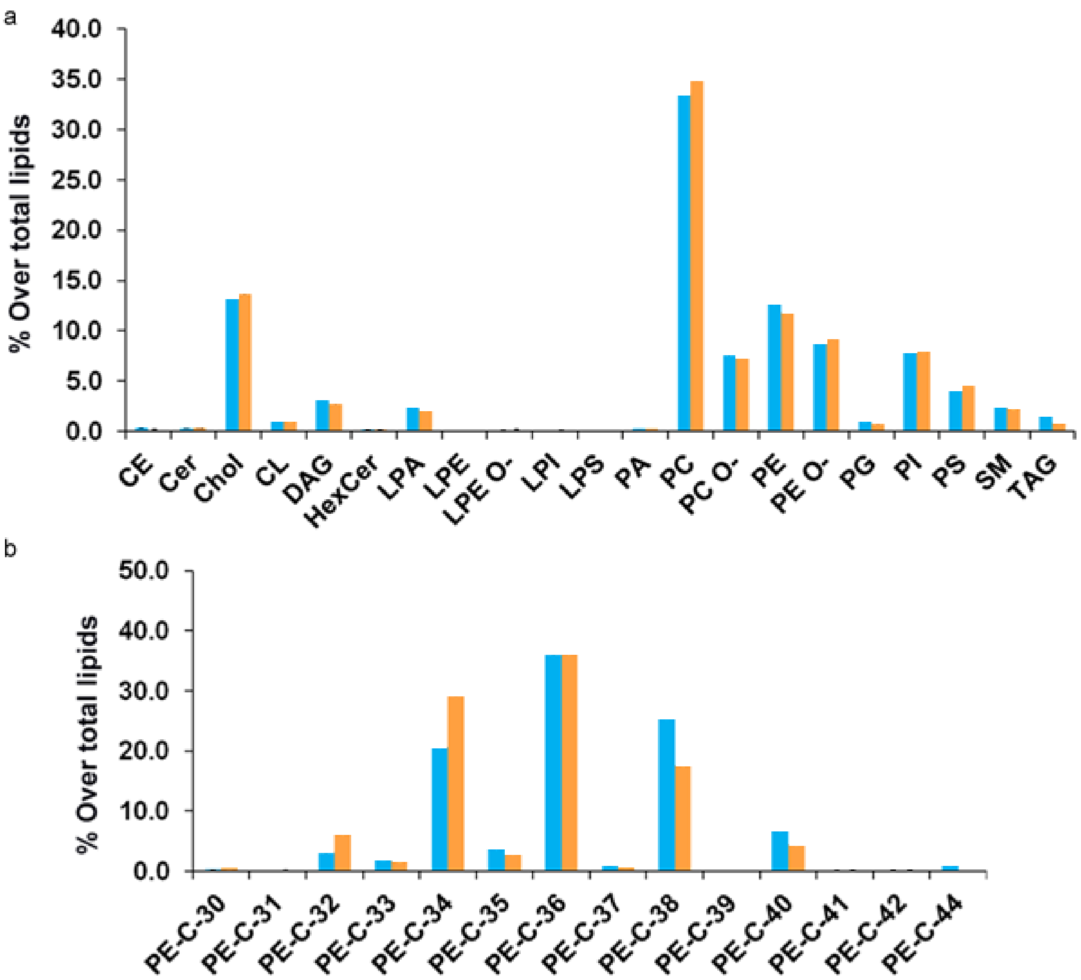

Supplementary Figure 5: Cell membrane analysis. A) Relative cell membrane lipids content expressed as percent of the total lipids. Blue bars represent the ETNK1-WT line, the orange ones represent the ETNK1-N244S. n=2 biologically independent samples. The error bars represent the standard deviation of the mean. CE=Cholesterol esters; CER=Ceramide; CHOL=Cholesterol; CL=Cardiolipin; DAG=Diacylglycerol; HexCer=Hexosylceramide; LPA=lyso-Phosphatidate; LPE=lyso-Phosphatidylethanolamine; LPE(O-)=lyso-Phosphatidylethanolamine (-ether); LPI=lyso-Phosphatidylinositol; LPS=lyso-Phosphatidylserine; PA=Phosphatidate; PC=Phosphatidylcholine; PC(O-)=Phosphatidylcholine (-ether); PE=Phosphatidylethanolamine; PE(O-)=Phosphatidylethanolamine (-ether); PG=Phosphatidylglycerol; PI=Phosphatidylinositol; PS=Phosphatidylserine; SM=Sphingomyelin; TAG=Triacylglycerol. B) Phosphatidylethanolamine carbon length profile, analyzed from C30 to C44, expressed as percent of the total lipids. Blue bars represent the ETNK1-WT line, the orange ones represent the ETNK1-N244S. n=2 biologically independent samples. The error bars represent the standard deviation of the mean. Source data are provided as a Source Data file.

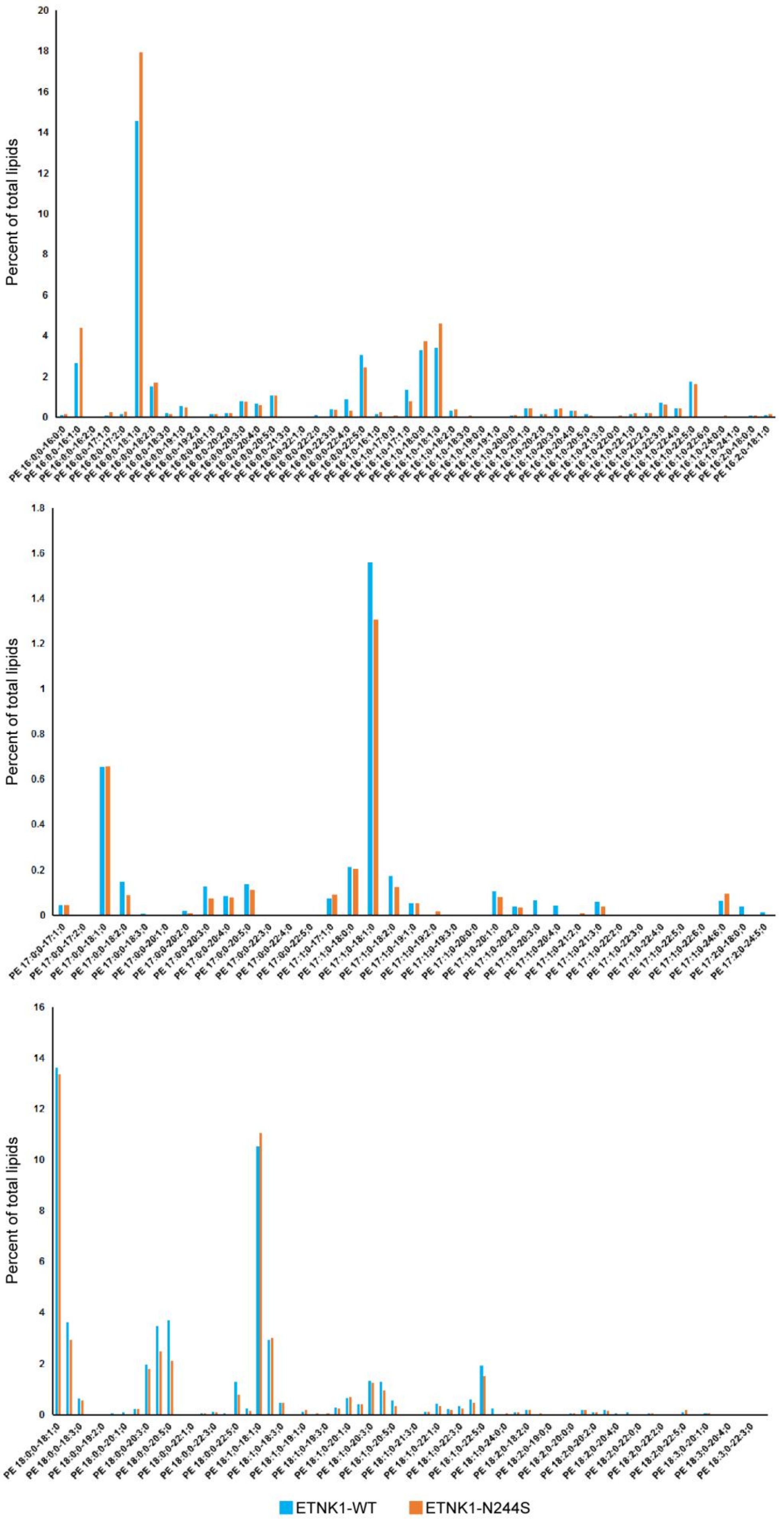
Percent of total lipids
Percent of total lipids
Percent of total lipids
ETNK1-WT
ETNK1-N244S

Supplementary Figure 6: Cell membrane phosphatidylethanolamine content. Phosphatidylethanolamine carbon length profile, analyzed from C12 to C24 and shown here from C16 to C18, is expressed as percent of the total lipids. Lipid species are annotated according to their molecular composition as: ‘lipid name’ followed by <Sum of the carbon atoms in the hydrocarbon moiety – fatty acid #1>:<sum of the double bonds in the hydrocarbon moiety – fatty acid #1>;<sum of hydroxyl groups - fatty acid #1>-<Sum of the carbon atoms in the hydrocarbon moiety – fatty acid #2>:<sum of the double bonds in the hydrocarbon moiety – fatty acid #2>;<sum of hydroxyl groups - fatty acid #2>. Blue bars represent the ETNK1-WT line, the orange ones represent the ETNK1-N244S. n=2 biologically independent samples. The error bars represent the standard deviation of the mean. Source data are provided as a Source Data file.

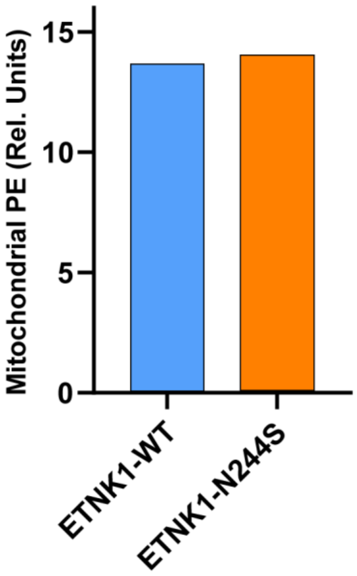

Supplementary Figure 7: Mitochondrial PE content. The boxplot represents the mitochondrial membrane content of PE. The left box represents the ETNK1-WT line, whereas the right one represents ETNK1-N244S (n=2). Source data are provided as a Source Data file.

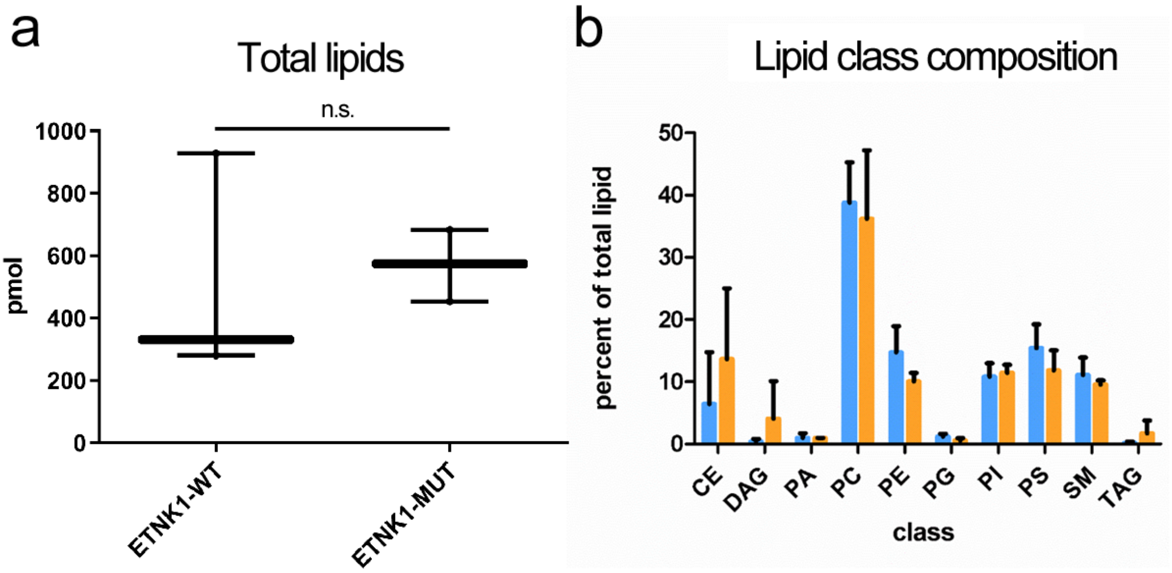

Supplementary Figure 8: Lipid content in patients' cells. A) Total lipids measured in patient samples. The left box represents the ETNK1-WT patients (n=3 biologically independent samples), whereas the right one represents the ETNK1-mutated ones (n=3 biologically independent samples). The boxplots delimit the interquartile range; the central bar represents the median; the whiskers extend from minimum to maximum. B) Relative cell lipids content expressed as percent of the total lipids. Blue bars represent the ETNK1-WT patients (n=3 biologically independent samples), the orange ones represent the ETNK1-mutated ones (n=3 biologically independent samples). The error bars represent the standard deviation of the mean. CE=Cholesterol esters; DAG=Diacylglycerol; PA=Phosphatidate; PC= Phosphatidylcholine; PE=Phosphatidylethanolamine; PG=Phosphatidylglycerol; PI=Phosphatidylinositol; PS=Phosphatidylserine; SM=Sphingomyelin; TAG=Triacylglycerol. Source data are provided as a Source Data file.

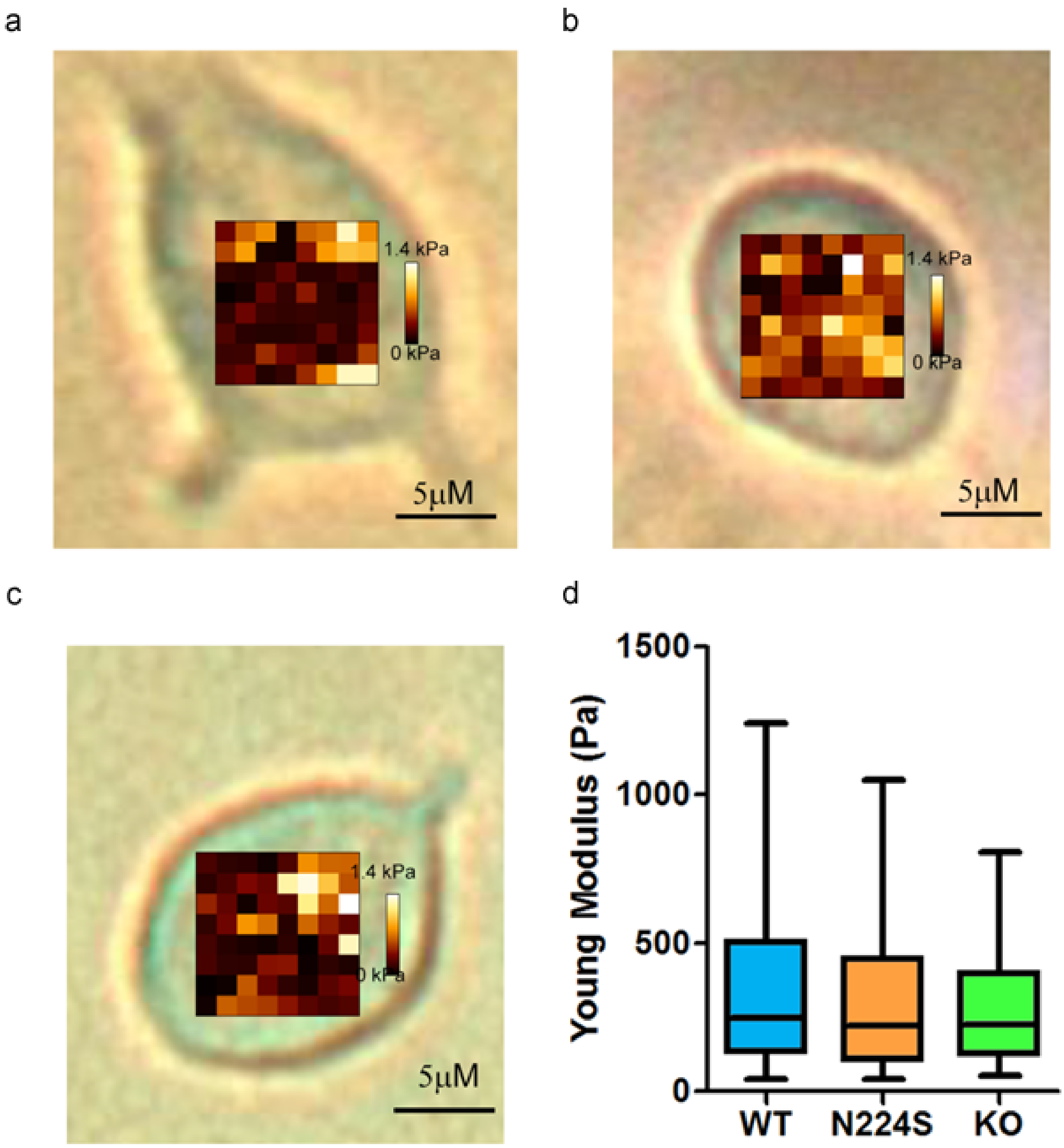

Supplementary Figure 9: Atomic force microscopy. A-C) Exemplificative atomic force microscopy force maps (8x8 pixels grid) of ETNK1-WT (A), ETNK1-N244S (B), and ETNK1-KO (C) cells. Pixel color code is scaled between 0 (black) and 1.4 kPa (white). D) Young's modulus of elasticity calculated for ETNK1-WT (blue), N244S (orange), and KO (green) lines. Boxes represent the interquartile range; whiskers delimit the 5th and 95th percentile range. n=3 biologically independent samples.

In order to probe locally the stiffness of the cells, the atomic force microscope (AFM) tip exerts a controlled force on the cell surface as a nano-indenter, and the deformation of the sample is detected. During a force measurement, the cantilever is moved towards the sample surface, while the cantilever vertical deflection, i.e. the applied force, is recorded (force curve). As the tip reaches the surface, a force is applied on the sample. As the force value increases, the sample is compressed and can be deformed both elastically and plastically. The softer the sample, the larger

the deformation recorded. At a specific fixed maximum force value (force set point), the cantilever retracts to reach the initial rest position and the pressure is released from the sample surface.

The evaluation of cell elastic properties, described quantitatively through the Young's modulus proportional to the ratio between the applied force and the deformation, was obtained by fitting the force curves with the Hertz-Sneddon model for contact mechanics applied to a paraboloidal tip. Source data are provided as a Source Data file.

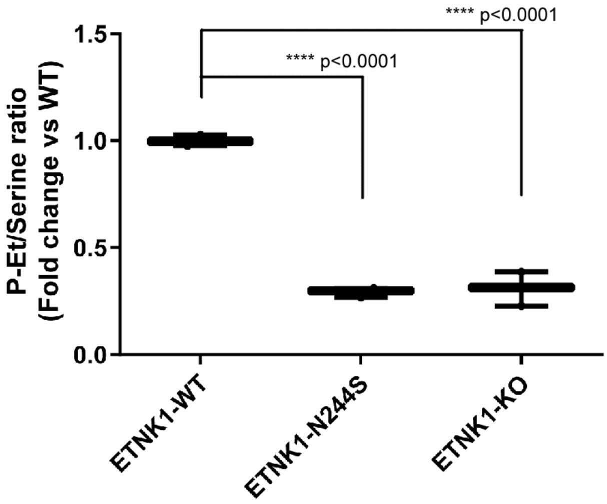

Supplementary Figure 10: P-Et content. Intracellular phosphoethanolamine/serine ratio in ETNK1-WT, N244S, and KO lines. The boxplots delimit the interquartile range; the central bar represents the median; the whiskers extend from minimum to maximum (n=3 biologically independent samples). Statistical analyses were performed using one-way ANOVA with Tukey's post-hoc test ($p<0.0001$ for ETNK1-WT vs N244S and for ETNK1-WT vs KO). Source data are provided as a Source Data file.

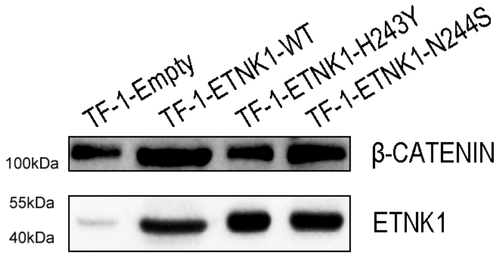

Supplementary Figure 11: Western blot done on the myeloid TF-1 cell line showing the expression of ETNK1 in cells transduced with an empty vector (TF-1-Empty), or with vectors encoding WT (TF-1-ETNK1-WT) or mutated (TF-1-ETNK1-H243Y and TF-1-ETNK1-N244S) ETNK1. Gel loading was normalized with β-Catenin. The experiment was performed once.

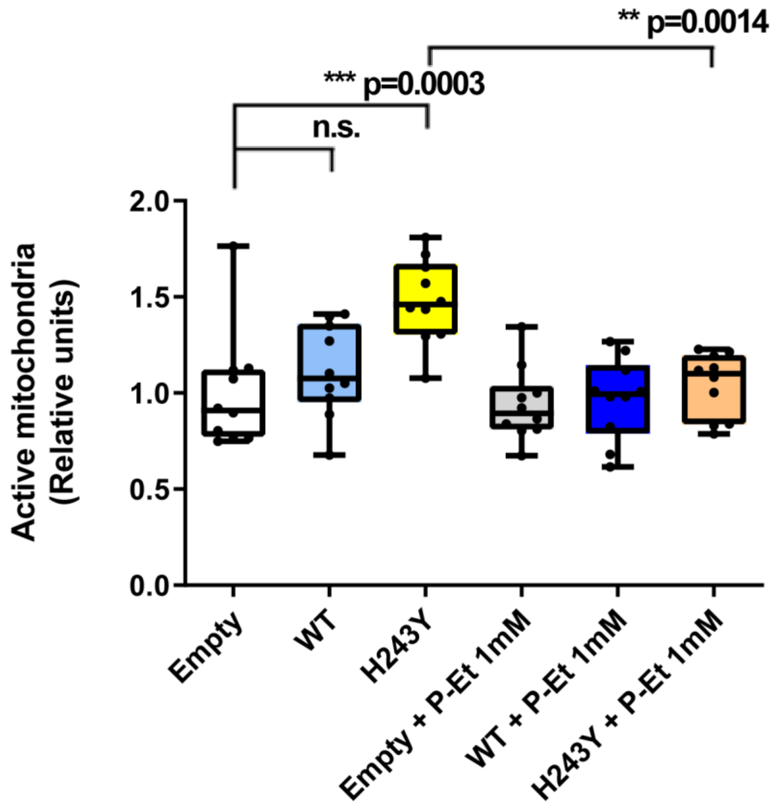

Supplementary Figure 12: Mitochondrial activity in the absence and presence of P-Et. Boxplots showing the MitoTracker Red-to-Green signal of TF-1-Empty, TF-1-ETNK1-WT, and TF-1-ETNK1-H243Y cell lines in the absence and presence of 1mM P-Et. The boxplots delimit the interquartile range; the central bar represents the median; the whiskers extend from minimum to maximum (n=10 representative fields). Statistical analyses were performed using one-way ANOVA with Tukey's post-hoc test. At least 200 cells were analyzed. Source data are provided as a Source Data file.

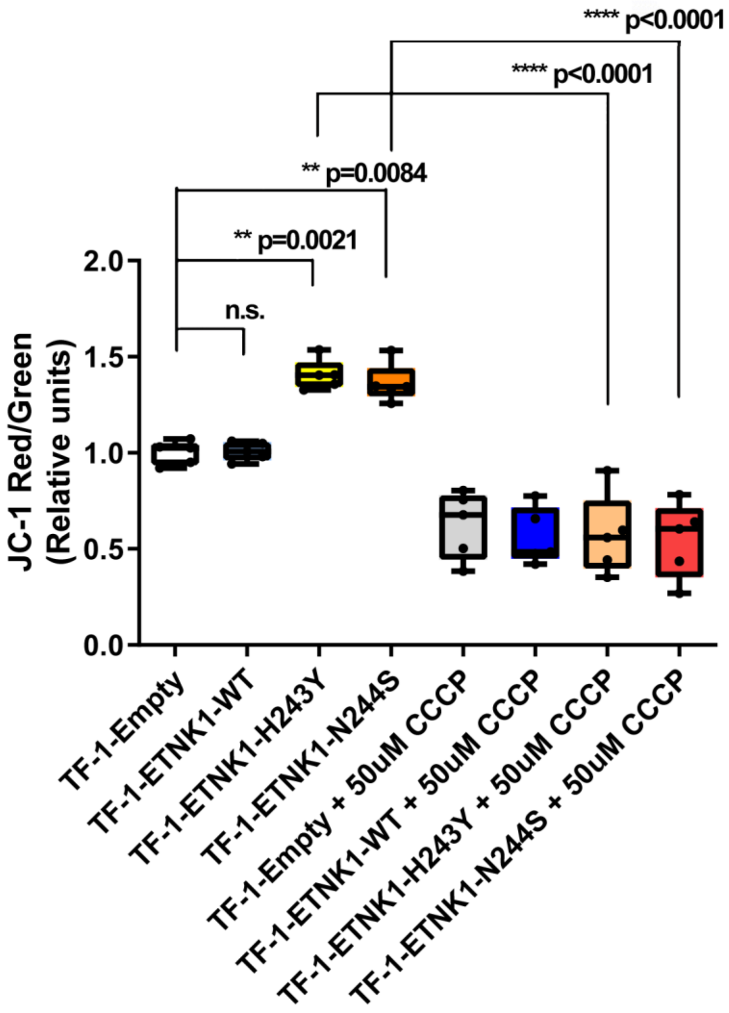

Supplementary Figure 13: Mitochondrial activity. Boxplots showing the JC-1 red-to-green signal ratio of TF-1-Empty, TF-1-ETNK1-WT, TF-1-ETNK1-H243Y, and TF-1-ETNK1-N244S cell lines normalized on TF-1-Empty in the absence and presence of 50µM CCCP. The boxplots delimit the interquartile range; the central bar represents the median; the whiskers extend from minimum to maximum (n=5 representative fields). Statistical analyses were performed using one-way ANOVA with Tukey's post-hoc test. At least 200 cells were analyzed. Source data are provided as a Source Data file.

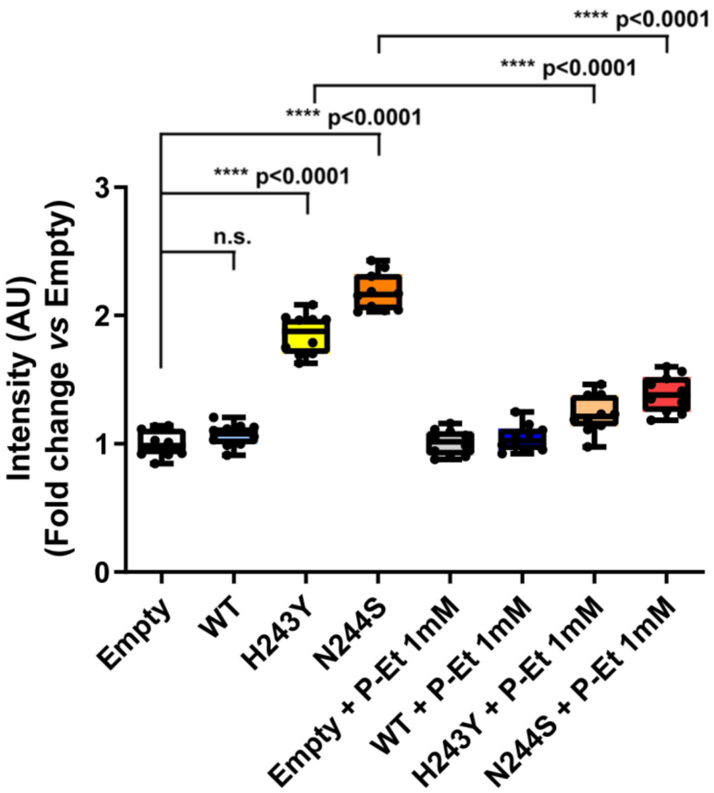

Supplementary Figure 14: ROS production. Intracellular reactive oxygen species as assessed by confocal microscopy using the CellROX reagent. ROS analysis was performed in TF-1-Empty, TF-1-ETNK1-WT, TF-1-ETNK1-H243Y, and TF-1-ETNK1-N244S in the absence and presence of P-Et 1mM. The boxplots delimit the interquartile range; the central bar represents the median; the whiskers extend from minimum to maximum (n=10 representative fields). Statistical analyses were performed using one-way ANOVA with Tukey's post-hoc test. At least 200 cells were analyzed. Source data are provided as a Source Data file.

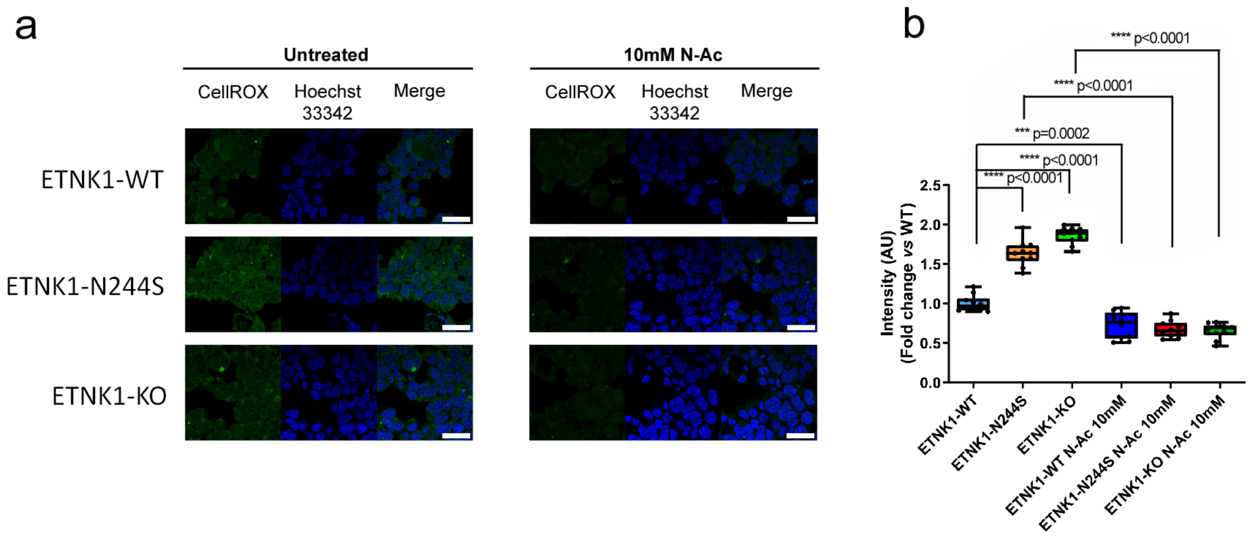

Supplementary Figure 15: ROS production. A) Intracellular reactive oxygen species as assessed by confocal microscopy using the CellROX reagent. ROS analysis was performed in ETNK1-WT, N244S, and KO lines in the absence and presence of 10mM N-Acetyl-L-cysteine (N-Ac). At least 200 cells were analyzed. The scale bar corresponds to 40µm. B) ROS quantification in ETNK1-WT, N244S, and KO lines in the absence and presence of 10mM N-Acetyl-L-cysteine (N-Ac). The boxplots delimit the interquartile range; the central bar represents the median; the whiskers extend from minimum to maximum (n=10 representative fields). Statistical analyses were performed using one-way ANOVA with Tukey's post-hoc test. Source data are provided as a Source Data file.

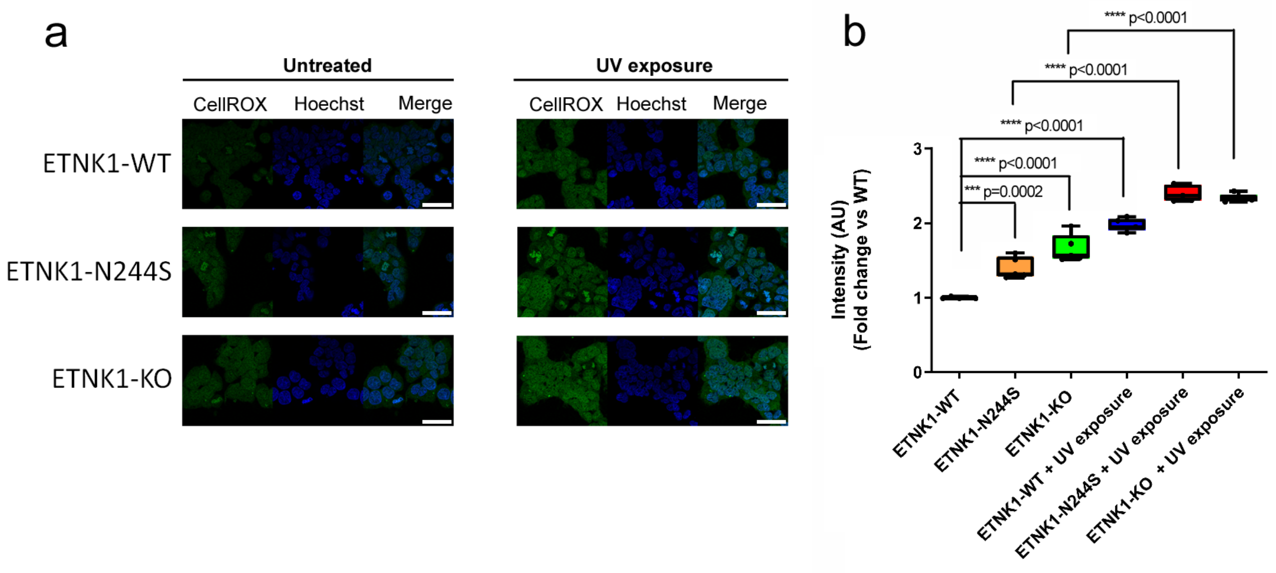

Supplementary Figure 16: ROS production. A) Intracellular reactive oxygen species as assessed by confocal microscopy using the CellROX reagent. ROS analysis was performed in ETNK1-WT, N244S, and KO lines after UV exposure compared to unexposed cells. At least 200 cells were analyzed. The scale bar corresponds to 40µm. B) ROS quantification in ETNK1-WT, N244S, and KO lines after UV exposure compared to unexposed cells. The boxplots delimit the interquartile range; the central bar represents the median; the whiskers extend from minimum to maximum (n=5 representative fields). Statistical analyses were performed using one-way ANOVA with Tukey's post-hoc test. Source data are provided as a Source Data file.

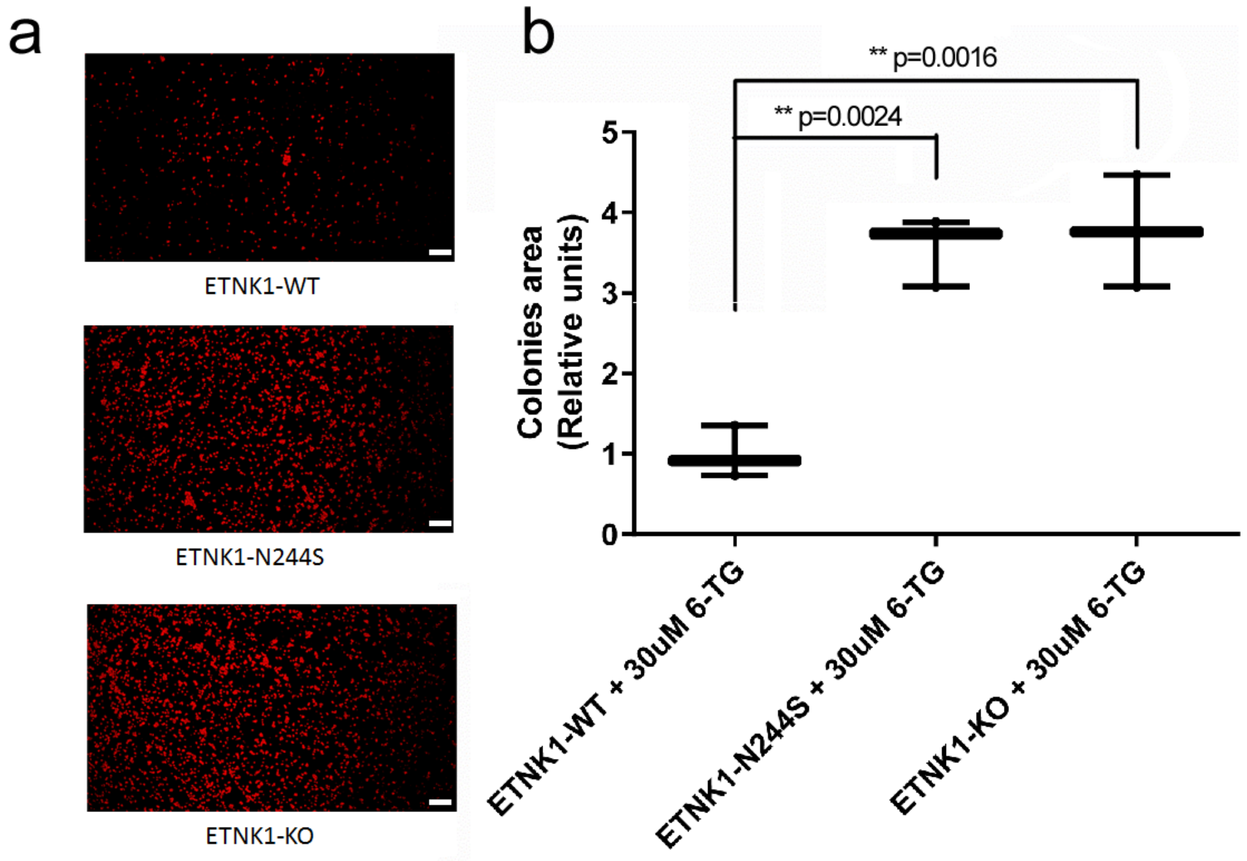

Supplementary Figure 17: 6-thioguanine resistance. A) Representative colony images of ETNK1-WT, N244S, and KO CRISPR cells in the presence of 30μM 6-TG. 1 million of cells was plated and exposed to 30μM of 6-TG for 15 days, while 1500 cells were plated for 15 days, as control. The experiment was performed in triplicate. The scale bar corresponds to 200μm.B) Boxplots showing the colony area of ETNK1-WT, N244S, and KO cell lines treated with 30μM 6-TG and normalized on ETNK1-WT. The boxplots delimit the interquartile range; the central bar represents the median; the whiskers extend from minimum to maximum (n=3 biologically independent samples). Statistical analyses were performed using one-way ANOVA with Tukey’s post-hoc test. Source data are provided as a Source Data file.

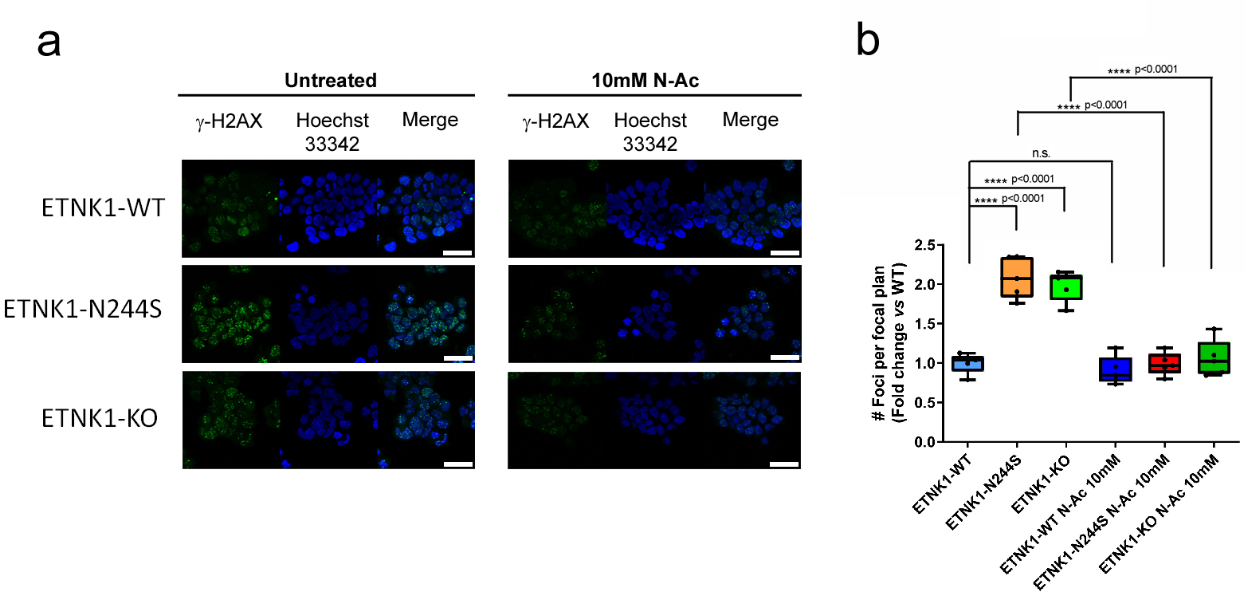

Supplementary Figure 18: Double-strand DNA damage. A) Confocal microscopy analysis of γH2AX foci in ETNK1-WT, N244S, and KO lines in the absence or presence of 10mM N-Acetyl-L-cysteine (N-Ac). At least 200 cells were analyzed in a single experiment. The scale bar corresponds to 40μm. B) γH2AX signal quantification in ETNK1-WT, N244S, and KO lines in the absence or presence of 10mM N-Acetyl-L-cysteine (N-Ac). The boxplots delimit the interquartile range; the central bar represents the median; the whiskers extend from minimum to maximum (n=5 representative fields). Statistical analyses were performed using one-way ANOVA with Tukey's post-hoc test. Source data are provided as a Source Data file.

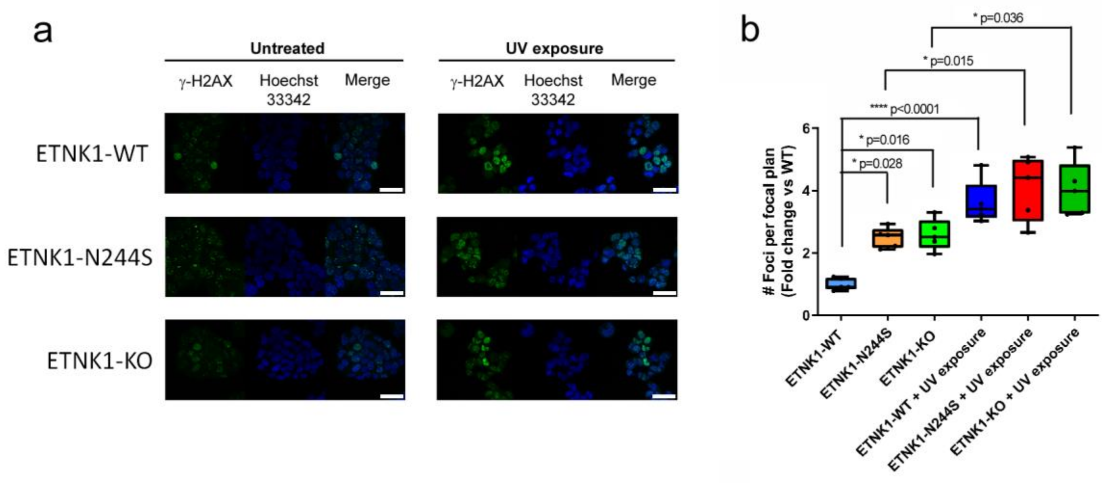

Supplementary Figure 19: Double-strand DNA damage. A) Confocal microscopy analysis of γH2AX foci in ETNK1-WT, N244S, and KO lines after UV exposure compared to unexposed cells. At least 200 cells were analyzed in a single experiment. The scale bar corresponds to 40µm. B) γH2AX signal quantification in ETNK1-WT, N244S, and KO lines after UV exposure compared to unexposed cells. The boxplots delimit the interquartile range; the central bar represents the median; the whiskers extend from minimum to maximum (n=5 representative fields). Statistical analyses were performed using one-way ANOVA with Tukey's post-hoc test. Source data are provided as a Source Data file.

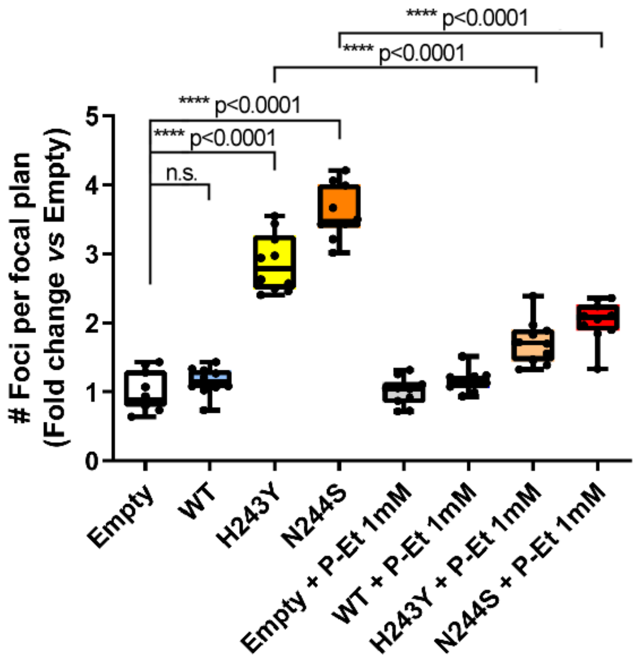

Supplementary Figure 20: Double-strand DNA damage. γH2AX signal quantification in TF-1-Empty, TF-1-ETNK1-WT, TF-1-ETNK1-H243Y, and TF-1-ETNK1-N244S lines in the absence or presence of 1mM P-Et. The boxplots delimit the interquartile range; the central bar represents the median; the whiskers extend from minimum to maximum (n=10 representative fields). Statistical analyses were performed using one-way ANOVA with Tukey's post-hoc test. At least 200 cells were analyzed. Source data are provided as a Source Data file.

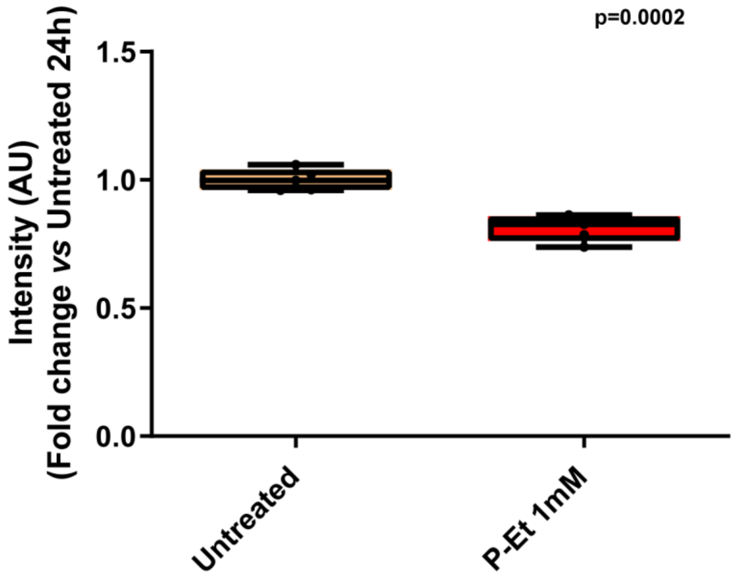

Supplementary Figure 21: ROS production. Intracellular reactive oxygen species as assessed by confocal microscopy using the CellROX reagent. ROS analysis was performed in primary bone marrow cells of aCML patient Pt 042 (ETNK1-N244S+) in the absence and presence of P-Et 1mM. The boxplots delimit the interquartile range; the central bar represents the median; the whiskers extend from minimum to maximum (n=5 representative fields). Statistical analyses were performed using a two-sided t-test. At least 200 cells were analyzed. Source data are provided as a Source Data file.

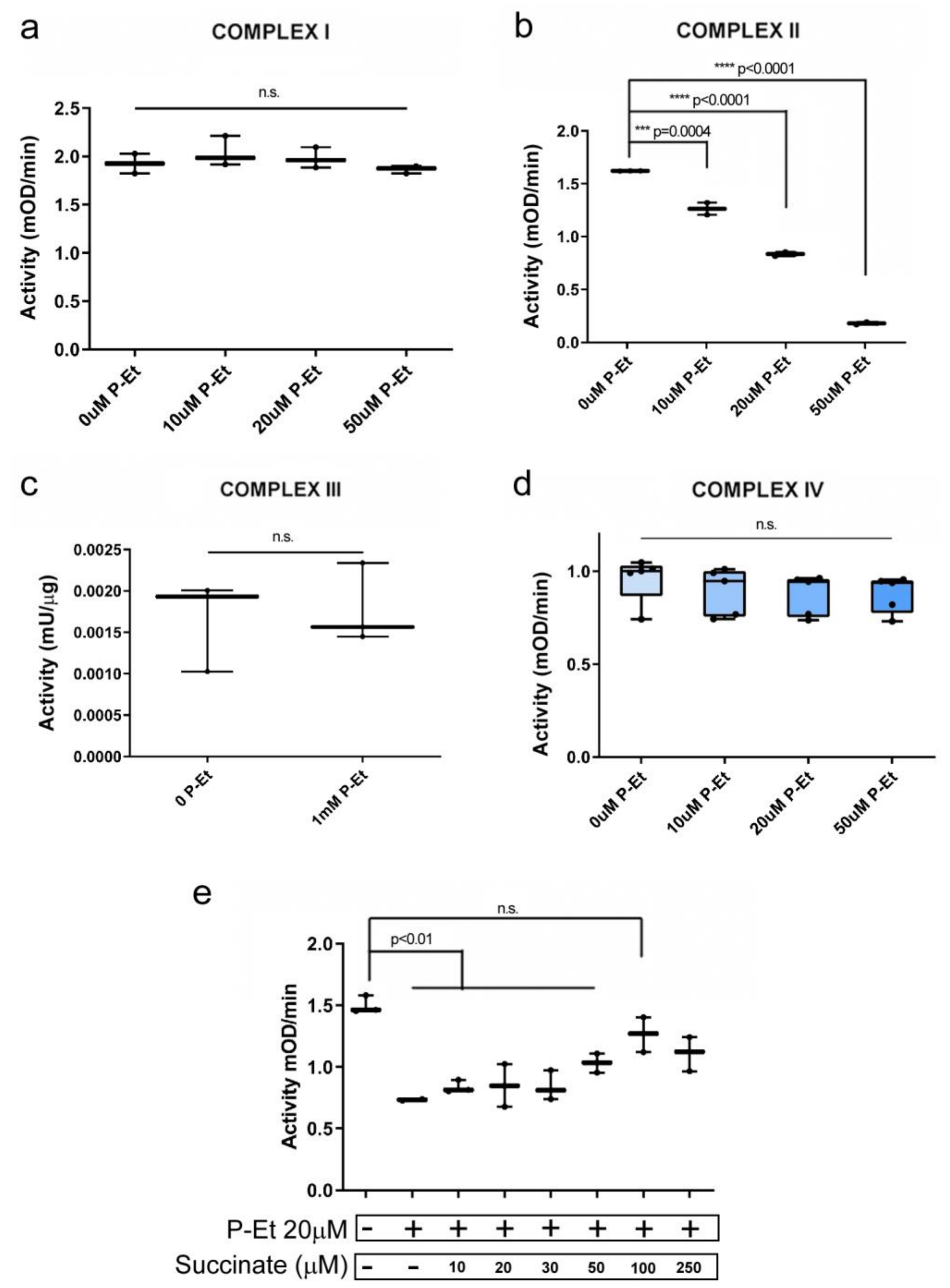

Supplementary Figure 22: Mitochondria complexes activity on TF-1 cells. A-D) Activity of mitochondria complexes I to IV in the presence of increasing concentrations of P-Et. The analysis of complexes I (n=3 independent replicates), II (n=3 independent replicates), and IV (n=5 independent replicates) was performed on mitochondria lysates, while the analysis of complex III (n=3) was performed on intact isolated mitochondria. The boxplots delimit the interquartile range; the central bar represents the median; the whiskers extend from minimum to maximum. Statistical analyses were performed using one-way ANOVA with Tukey's post-hoc test (A,B,D) and t-test (C). E) The activity of mitochondria complex II was assessed in the absence and presence of 20µM P-Et in combination with increasing concentrations of succinate. The boxplots delimit the interquartile range; the central bar represents the median; the whiskers extend from minimum to maximum (n=3 independent replicates). Statistical analyses were performed using one-way ANOVA with Tukey's post-hoc test. Source data are provided as a Source Data file.
