# Supplementary material for: ETNK1 mutations induce a mutator phenotype that can be reverted with phosphoethanolamine
Source: Nat Commun. 2020 Nov 23;11:5938. doi: 10.1038/s41467-020-19721-w (PMC7684297; doi:10.1038/s41467-020-19721-w)
Supplement: Supplementary file 3 — Description of Additional Supplementary Files [file 41467_2020_19721_MOESM3_ESM.pdf]

Description of Additional Supplementary Files

Supplementary Data 1: Oligonucleotides list. The gRNA and the ssODN sequences used for CRISPR/Cas9 genome editing, together with the primers used for sequencing are reported.

Supplementary Data 2: Abbreviations used in Supplementary Figure 4.

Supplementary Data 3: RNA-Seq data. The list of the 119 differentially expressed genes (FDR<0.1) between ETNK1-WT and ETNK1-N244S cell lines are reported. Statistical analyses were performed using the DESeq2 package; Benjamini Hochberg correction was used in order to control the false discovery rate.

Supplementary Data 4: 6-thioguanine assay. The number of ETNK1-WT, ETNK1-N244S, and ETNK1-KO colonies grown in the presence/absence of 6-thioguanine in three different experiments are listed, together with the mean and standard deviation.

Supplementary Data 5: 6-thioguanine assay after P-Et or tigecycline treatment. The number of ETNK1-WT, ETNK1-N244S, and ETNK1-KO colonies grown in the presence/absence of 6-thioguanine in three different experiments after exposure to P-Et 1mM or tigecycline 2.5μM for 15 days are listed, together with the mean and standard deviation.

Supplementary Data 6: Somatic variants identified by matched exome sequencing in atypical Chronic Myeloid Leukemia patient 004. Somatic variants identification was performed using a dedicated algorithm.

Supplementary Data 7: Somatic variants identified by matched exome sequencing in atypical Chronic Myeloid Leukemia patient 037. Somatic variants identification was performed using a dedicated algorithm.

Supplementary Data 8: Somatic variants identified by matched exome sequencing in atypical Chronic Myeloid Leukemia patient 014. Somatic variants identification was performed using a dedicated algorithm.

Supplementary Data 9: Somatic variants identified by matched exome sequencing in atypical Chronic Myeloid Leukemia patient 038. Somatic variants identification was performed using a dedicated algorithm.

Supplementary Data 10: Somatic variants identified by matched exome sequencing in atypical Chronic Myeloid Leukemia patient 035. Somatic variants identification was performed using a dedicated algorithm.

Supplementary Data 11: Somatic variants identified by matched exome sequencing in atypical Chronic Myeloid Leukemia patient 025. Somatic variants identification was performed using a dedicated algorithm.

Supplementary Data 12: Somatic variants identified by matched exome sequencing in atypical Chronic Myeloid Leukemia patient 019. Somatic variants identification was performed using a dedicated algorithm.

Supplementary Data 13: Somatic variants identified by matched exome sequencing in atypical Chronic Myeloid Leukemia patients 013. Somatic variants identification was performed using a dedicated algorithm.

Supplementary Data 14: Somatic variants identified by matched exome sequencing in atypical Chronic Myeloid Leukemia patient 042. Somatic variants identification was performed using a dedicated algorithm.

Supplementary Data 15: Antibodies list. The name, clone, brand, code, and dilution of used antibodies for each assay are reported.
