# Supplementary material for: ETNK1 mutations induce a mutator phenotype that can be reverted with phosphoethanolamine
Source: Nat Commun. 2020 Nov 23;11:5938. doi: 10.1038/s41467-020-19721-w (PMC7684297; doi:10.1038/s41467-020-19721-w)
Supplement: Supplementary file 19 — Reporting summary [file 41467_2020_19721_MOESM19_ESM.pdf]

nature research

Corresponding author(s): Rocco Piazza

Last updated by author(s): Sep 29, 2020

Reporting Summary

Nature Research wishes to improve the reproducibility of the work that we publish. This form provides structure for consistency and transparency in reporting. For further information on Nature Research policies, see our Editorial Policies and the Editorial Policy Checklist.

Statistics

For all statistical analyses, confirm that the following items are present in the figure legend, table legend, main text, or Methods section.

n/a	Confirmed	
☐	☒	The exact sample size (n) for each experimental group/condition, given as a discrete number and unit of measurement
☐	☒	A statement on whether measurements were taken from distinct samples or whether the same sample was measured repeatedly
☐	☒	The statistical test(s) used AND whether they are one- or two-sided<br>*Only common tests should be described solely by name; describe more complex techniques in the Methods section.*
☐	☒	A description of all covariates tested
☐	☒	A description of any assumptions or corrections, such as tests of normality and adjustment for multiple comparisons
☐	☒	A full description of the statistical parameters including central tendency (e.g. means) or other basic estimates (e.g. regression coefficient) AND variation (e.g. standard deviation) or associated estimates of uncertainty (e.g. confidence intervals)
☐	☒	For null hypothesis testing, the test statistic (e.g. F, t, r) with confidence intervals, effect sizes, degrees of freedom and P value noted<br>*Give P values as exact values whenever suitable.*
☒	☐	For Bayesian analysis, information on the choice of priors and Markov chain Monte Carlo settings
☒	☐	For hierarchical and complex designs, identification of the appropriate level for tests and full reporting of outcomes
☒	☐	Estimates of effect sizes (e.g. Cohen's d, Pearson's r), indicating how they were calculated

Our web collection on statistics for biologists contains articles on many of the points above.

Software and code

Policy information about availability of computer code

Data collection	Mitochondrial respiratory data were recorded with DatLab 6 software.
Data analysis	Statistical analyses were performed with GraphPad prism 8.0.2. Mitochondrial respiration was analyzed with DatLab 6 software; Confocal microscopy images were acquired with ZEN 2.3 black edition Software and processed with ImageJ version 1.53c vith Java 1.8.0_66 (64 bit). Mass spectrometry lipidomics data were processed with LipidXplorer v 1.2. Targeted mass spectrometric metabolite profiling data were processed using MassLynx v 4.1. OxoGuanineProfiler v 1.0 was used to process oxoguanine data; OxoGuanineProfiler is available in the source data file.

For manuscripts utilizing custom algorithms or software that are central to the research but not yet described in published literature, software must be made available to editors and reviewers. We strongly encourage code deposition in a community repository (e.g. GitHub). See the Nature Research guidelines for submitting code & software for further information.

Data

Policy information about availability of data

All manuscripts must include a data availability statement. This statement should provide the following information, where applicable:

- Accession codes, unique identifiers, or web links for publicly available datasets
- A list of figures that have associated raw data
- A description of any restrictions on data availability

The NGS data have been deposited in NCBI's Sequence Read Archive (SRA) and are accessible through accession number PRJNA501862 (https://www.ncbi.nlm.nih.gov/sra/?term=PRJNA501862).

Field-specific reporting

Please select the one below that is the best fit for your research. If you are not sure, read the appropriate sections before making your selection.

- [x] Life sciences
- [ ] Behavioural & social sciences
- [ ] Ecological, evolutionary & environmental sciences

For a reference copy of the document with all sections, see nature.com/documents/nr-reporting-summary-flat.pdf

Life sciences study design

All studies must disclose on these points even when the disclosure is negative.

Sample size	Given the rarity of the aCML samples, all available specimens were used in this study.
Data exclusions	No data were excluded from the analysis.
Replication	We replicated all the experiments in two different cell lines. The replication was successful.
Randomization	As we are studying the effect of a specific somatic mutations in terms of downstream signalling, randomization is not relevant to this work.
Blinding	As we are studying the effect of a specific somatic mutations in terms of downstream signalling, blinding is not relevant to this work.

Reporting for specific materials, systems and methods

We require information from authors about some types of materials, experimental systems and methods used in many studies. Here, indicate whether each material, system or method listed is relevant to your study. If you are not sure if a list item applies to your research, read the appropriate section before selecting a response.

Materials & experimental systems

n/a	Involved in the study	
[ ]	[x]	Antibodies
[ ]	[x]	Eukaryotic cell lines
[x]	[ ]	Palaeontology and archaeology
[x]	[ ]	Animals and other organisms
[ ]	[x]	Human research participants
[ ]	[x]	Clinical data
[x]	[ ]	Dual use research of concern

Methods

n/a	Involved in the study	
[ ]	[x]	ChIP-seq
[x]	[ ]	Flow cytometry
[x]	[ ]	MRI-based neuroimaging

Antibodies

Antibodies used

Phospho-Histone H2A.X (Ser139) (20E3) Cell Signaling Technology 9718
ETNK1 [N2C3] GeneTex GTX105887
Anti-8-Oxoguanine Antibody, clone 483.15 Millipore-Merck
Anti-ß-Catenin Clone 14/Beta-Catenin BD 610153
Anti-Histone H3 Abcam 1791
Anti-Actin Sigma A2066
Alexa 488-conjugated secondary antibody Thermo Fisher Scientific A-11008
Anti-rabbit Bio-Rad 1706515
Anti-mouse Bio-Rad 1706516

Validation

- Phospho-Histone H2A.X (Ser139) (20E3) Cell Signaling Technology 9718 was validated in Human, Monkey, Mouse, Rat for Western Blotting, Immunohistochemistry, Immunofluorescence, Flow Cytometry
- ETNK1 [N2C3] GeneTex GTX105887 was validated in Human and Mouse for WB and IHC-P.
- Anti-8-Oxoguanine Antibody, clone 483.15 Millipore-Merck was validated in Human, Mouse, Rat and Monkey for ELISA, Immunocytochemistry, Chromatin Immunoprecipitation.
- Anti-ß-Catenin Clone 14/Beta-Catenin BD 610153 was validated in Human, Mouse, Rat, Dog and Chicken for Western blot, Immunohistochemistry, Immunoprecipitation and Immunofluorescence.

Eukaryotic cell lines

Policy information about cell lines

Cell line source(s)	HEK-293-Flp-In were purchased from Thermo Fisher Scientific. TF-1 were purchased from DSMZ.
Authentication	Authentication was performed by Thermo Fisher and DSMZ by multiplex PCR of minisatellite markers, revealing a unique DNA profile.
Mycoplasma contamination	All cell lines tested negative for mycoplasma.
Commonly misidentified lines (See ICLAC register)	No commonly misidentified lines were used in this study.

Human research participants

Policy information about studies involving human research participants

Population characteristics	Patients were recruited if > 18 age and in presence of a diagnosis of atypical chronic myeloid leukemia.
Recruitment	Participants were recruited in presence of a diagnosis of atypical chronic myeloid leukemia and divided in ETNK1-positive in presence of somatic ETNK1 variants occurring at nucleotides 243, 244 or 245 (i.e. ETNK1 mutational hotspot) and ETNK1-negative in absence of ETNK1 somatic mutations.
Ethics oversight	The ethic committee of the University of Milano - Bicocca

Note that full information on the approval of the study protocol must also be provided in the manuscript.

Clinical data

Policy information about clinical studies

All manuscripts should comply with the ICMJEguidelines for publication of clinical research and a completedCONSORT checklist must be included with all submissions.

Clinical trial registration	No clinical trial is present in this study
Study protocol	As the purpose of this study is the analysis of the downstream signalling in ETNK1-mutated patients, no specific study protocol is defined.
Data collection	Samples were collected starting from January 2009
Outcomes	As the purpose of this study is the analysis of the downstream signalling in ETNK1-mutated patients, no specific outcome is defined.

ChIP-seq

Data deposition

- [x] Confirm that both raw and final processed data have been deposited in a public database such as GEO.
- [x] Confirm that you have deposited or provided access to graph files (e.g. BED files) for the called peaks.

Data access links<br>*May remain private before publication.*	https://www.ncbi.nlm.nih.gov/sra/?term=PRJNA501862<br>https://osf.io/fd3m2
Files in database submission	https://www.ncbi.nlm.nih.gov/sra/?term=PRJNA501862<br>WT-ChIP-1_R1_001.fastq.gz<br>WT-ChIP-1_R2_001.fastq.gz<br>WT-ChIP-2_R1_001.fastq.gz<br>WT-ChIP-2_R2_001.fastq.gz<br>N244S-ChIP-1_R1_001.fastq.gz<br>N244S-ChIP-1_R2_001.fastq.gz<br>N244S-ChIP-2_R1_001.fastq.gz<br>N244S-ChIP-2_R2_001.fastq.gz<br><br>https://osf.io/fd3m2<br>WT-ChIP-1.bed.gz<br>WT-ChIP-2.bed.gz<br>N244S-ChIP-1.bed.gz<br>N244S-ChIP-2.bed.gz<br>OxoguanineProfiler.zip

Genome browser session (e.g. UCSC)	No longer applicable

Methodology

Replicates	Two replicates were generated for both N244S and WT lines
Sequencing depth	ETNK1_N244S_1: 38268343 total reads; 32430242 mapped reads; 1446189 supplementary reads; 10665275 duplicate reads.<br>ETNK1_N244S_2: 32804096 total reads; 28368192 mapped reads; 1231690 supplementary reads; 5318341 duplicate reads.<br>ETNK1_WT_1: 21417147 total reads; 18219710 mapped reads; 816767 supplementary reads; 3774935 duplicate reads.<br>ETNK1_WT_2: 18919588 total reads; 15183666 mapped reads; 691946 supplementary reads; 2773281 duplicate reads.
Antibodies	Anti-8-Oxoguanine Antibody, clone 483.15 from Millipore-Merck was used for Immunoprecipitation.
Peak calling parameters	Reads non properly mapped, supplementary reads or reads with mapping quality lower than 20 were filtered out. Reads mapping to mitochondria chromosome were similarly discarded. The remaining reads were binned according to the mapping chromosome and to their first-base position in the reference genome, using 10000-bases bin size. All the bins were subsequently normalized for the total number of filtered reads and bins containing less than 10 hits were discarded. Finally, the total number of normalized hits per individual chromosome was calculated for two independent experiments and the mean value calculated.<br>A paired, per-chromosome Wilcoxon Signed Rank Test was then performed on the final case/control data with threshold p-value set at 0.05.
Data quality	Data quality was analyzed with FastQC as well as with Sam-Profiler.
Software	Raw sequencing data were initially aligned against the reference hg38 human genome (GCA_000001405.15_GRCh38) using the BWA-MEM algorithm. Reads were subsequently processed as described in the 'Peak calling parameters' section.
